# Gallbladder ascariasis in Kabul—focus on ultrasound and conservative therapy: a case report

**DOI:** 10.1093/omcr/omaf040

**Published:** 2025-05-28

**Authors:** Mohammad Sharif Sediqi, Mansoor Aslamzai, Abdulhakim Mukhlis, Khesrow Ekram

**Affiliations:** Department of Pediatrics, Kabul University of Medical Sciences, Kabul, Afghanistan; Department of Neonatology, Kabul University of Medical Sciences, Kabul, Afghanistan; Department of Neonatology, Kabul University of Medical Sciences, Kabul, Afghanistan; Department of Pediatrics, Kabul University of Medical Sciences, Kabul, Afghanistan

**Keywords:** Paediatrics, Gastroenterology, Hepatology, Radiology

## Abstract

The most frequent human intestinal nematode is *Ascaris lumbricoides*. Ascariasis is one of the most prevalent intestinal illnesses in underdeveloped nations, including Afghanistan. It affects over 25% of the world’s population and causes about 20 000 fatalities annually due to its unfavorable clinical course. In endemic locations, 50%–60% of pediatric admissions to surgical emergency rooms are caused by *A. lumbricoides.* Due to the cystic duct’s narrowness and tortuousness, migration of the worm to the gallbladder is less common than migration to the bile duct. When it does, acalculous cholecystitis is triggered. We report the case of a three year old girl presenting with abdominal pain, fever and vomiting. Laboratory tests revealed eosinophilia and elevated liver enzymes. Ultrasound examination identified a live *Ascaris* worm in the gallbladder. The patient was treated successfully with anti-helminthic therapy, resulting in symptom resolution.

## Introduction

The most frequent human intestinal nematode is *Ascaris lumbricoides*. Although it is widespread over the world, it is particularly common in the majority of tropical and subtropical climates. It affects over 25% of the world’s population [1] and causes about 20 000 fatalities annually due to its unfavorable clinical course. In endemic locations, 50%–60% of pediatric admissions to surgical emergency rooms are caused by *A. lumbricoides*. 10% of these admissions are due to hepatobiliary and pancreatic ascariasis [2] although the prognosis of *Ascaris* is often benign, it is linked to a number of problems, such as hepatobiliary ascariasis, intestinal blockage caused by a worm bolus, pancreatic ascariasis, acute appendicitis, peritoneal granulomas, small bowel volvulus, and small bowel intussusception. The frequency of ascariasis in Afghanistan is not well-documented. It is an uncommon condition, and our hospital does not currently have any data on this instance.

## Case report

**Figure 1:**
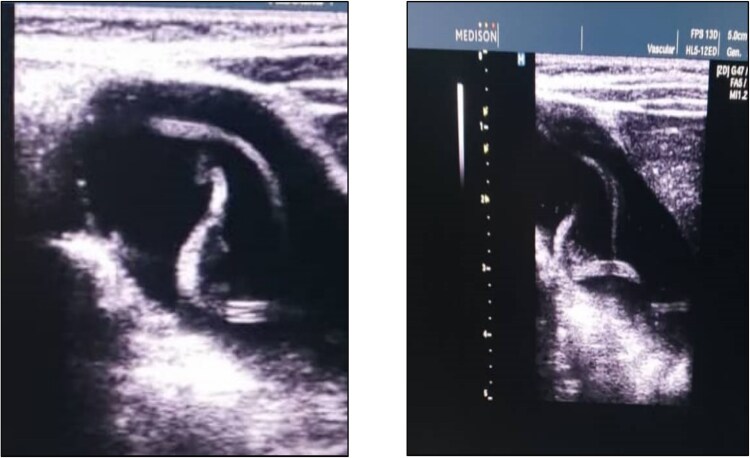
Ultrasound image of the patient shows *Ascaris* worm in gallbladder.

Here, we offer a case study of an Afghan youngster who visited our Department of Pediatrics this year for treatment of gallbladder ascariasis. A 3-year-old Afghan girl (36 months old) who had acute gastroenteritis arrived to our department with history of fever, vomiting, diarrhea and abdominal pain for last seven days. Fever was moderate in grade and continuous and vomiting was non-projectile and many times a day. She had 4–5 times of loose motions in a day and she had mild dehydration. Abdominal pain was localized to right upper quadrant area and revealed mild tender with palpation. Liver was normal in size and there was no organomegaly. She also had history of jaundice in last 5 months for which she was admitted in another hospital before this. During a physical examination, the patient was ill looking, a rectal temperature of 38.6°C, a respiratory rate of 36/min, a heart rate of 131/min, and 95% oxygen saturation level. Hemoglobin levels of 14 g/dl, total leukocyte count of 15 400/mm^3^ (polymorphs 63.3%, lymphocytes 17.4%, eosinophil 17%, and monocytes 2%), platelet count of 386 000/mm^3^, and C-reactive protein levels of 24 mg/dl were all found during blood tests. Her erythrocyte sedimentation rate was 45 mm during the 1 h, according to the laboratory analysis. Liver function tests showed ALT: 89 U/l, AST: 76 U/l, ALP: 320 U/l and Bilirubin levels were slightly raised. Eosinophils made up 17% of the total WBC count. Other tests were clear. In stool routine exam, *Ascaris* worm and ova were detected. A linear echogenic structure consistent with a live *Ascaris* worm was identified in the gallbladder without evidence of cholecystitis or obstruction. (Fig. 1). She was treated with oral mebendazole (100 mg twice daily for 3 days) and supportive care, including analgesics and hydration. No surgical intervention was required. Injection cefotaxime and amikacin was also administered because the inflammatory markers were increased. Dehydration was treated with oral rehydration therapy. The patients symptoms resolved within 72 h and the follow up ultrasound confirmed the absence of the worm. She was advised on hygiene practices to prevent reinfection.

## Discussion

Due to the gallbladder’s difficult access due to the tortuous and constrictive cystic duct, *Ascaris* worm infestation is uncommon [3, 4]. The gallbladder is obstructed by worms or their eggs in the common bile duct and cystic duct, which causes acute cholecystitis and gallbladder distension. The parameters that affect a parasite’s migration into the gallbladder include the patient’s age, sex, and worm load in the stomach [5]. Adult worms can partially or completely clog the intestines, causing symptoms such as abdominal pain, distension, colic, nausea, anorexia, and sporadic diarrhea. The patients will have nausea, vomiting, fever, and excruciating radiating pain if they develop cholangitis, pancreatitis, or appendicitis jaundice.

Eosinophilia on a total blood count may indicate helminthic infection. The diagnosis is dependent on a stool examination for parasites and ova, however it is not specific. Ascariasis is characterized by large, three-layered, brown eggs measuring 50 mm in size in patients. Clinically, serological testing for ascariasis is useless. Abdominal sonography is essential for determining the diagnosis of biliary ascariasis, despite the possibility that active live worms’ zigzag and meandering motions could misinterpret sonographic findings [6]. The presence in the gallbladder of a single, long, linear or curved echogenic structure without acoustic shadowing, resembling an anechogenic tube, and characteristic movement of these long echogenic structures within the bile duct are the characteristic sonographic features of worms in the bile duct.

Gallbladder distention, edema of the gallbladder wall, sludge inside the gallbladder, a coiled echogenic structure within the gallbladder, many liver abscesses, and edematous pancreatitis are other symptoms that are less specific or are an expression of the problems [7].

Except in cases where an accompanying condition is present or a problem develops, conservative treatment should be the initial course of action for gallbladder ascariasis [8–11]. Acceptable substitutes include mebendazole 100 mg twice daily for three days, 500 mg as a single dosage, and albendazole 400 mg given orally in a single dose. When ascariasis and whip-worm infection coexist in a patient, mebendazole is very appropriate [12, 13]. In our facility, it is the preferred kind of treatment due to its success. Surgery is only used when other treatments have failed and there is no spontaneous clearance of the worms, as well as when there is a dead worm inside the gallbladder and worm associated with calculi. These are extremely uncommon occurrences in our experience, and until this point, none of the patients needed surgery.

## Conclusion

Gallbladder ascariasis should be taken into consideration in all patients who come with severe radiating pain, colic, nausea, anorexia, and intermittent diarrhea along with jaundice. Eosinophilia in the blood count, the presence of ova and parasites in the stool, as well as a single, long, linear or curved echogenic structure that resembles an anechogenic tube without any acoustic shadowing and moves characteristically within the bile duct on abdominal ultrasound, are conclusive diagnostic indicators. The medicine mebendazole is efficient in treating the condition. Surgery is not frequently required.

## Consent

Guardians of the patients gave their permission for the case report to be published. Consideration was given to the Helsinki Declaration.

## Guarantor

Teaching Assistant Dr. Mohammad Sharif Sediqi.

## Data Availability

The corresponding author will provide the documents utilized in the current study upon reasonable request.
